# Organizing Effects of Sex Steroids on Brain Aromatase Activity in Quail

**DOI:** 10.1371/journal.pone.0019196

**Published:** 2011-04-29

**Authors:** Charlotte A. Cornil, Gregory F. Ball, Jacques Balthazart, Thierry D. Charlier

**Affiliations:** 1 GIGA Neurosciences, University of Liège, Liège, Belgium; 2 Department of Psychological and Brain Sciences, Johns Hopkins University, Baltimore, Maryland, United States of America; University of Akron, United States of America

## Abstract

Preoptic/hypothalamic aromatase activity (AA) is sexually differentiated in birds and mammals but the mechanisms controlling this sex difference remain unclear. We determined here (1) brain sites where AA is sexually differentiated and (2) whether this sex difference results from organizing effects of estrogens during ontogeny or activating effects of testosterone in adulthood. In the first experiment we measured AA in brain regions micropunched in adult male and female Japanese quail utilizing the novel strategy of basing the microdissections on the distribution of aromatase-immunoreactive cells. The largest sex difference was found in the medial bed nucleus of the stria terminalis (mBST) followed by the medial preoptic nucleus (POM) and the tuberal hypothalamic region. A second experiment tested the effect of embryonic treatments known to sex-reverse male copulatory behavior (i.e., estradiol benzoate [EB] or the aromatase inhibitor, Vorozole) on brain AA in gonadectomized adult males and females chronically treated as adults with testosterone. Embryonic EB demasculinized male copulatory behavior, while vorozole blocked demasculinization of behavior in females as previously demonstrated in birds. Interestingly, these treatments did not affect a measure of appetitive sexual behavior. In parallel, embryonic vorozole increased, while EB decreased AA in pooled POM and mBST, but the same effect was observed in both sexes. Together, these data indicate that the early action of estrogens demasculinizes AA. However, this organizational action of estrogens on AA does not explain the behavioral sex difference in copulatory behavior since AA is similar in testosterone-treated males and females that were or were not exposed to embryonic treatments with estrogens.

## Introduction

With the recognition that numerous mental disorders are sex-biased, the study of sex differences in the brain has now emerged as a field on its own [1]. Because reproduction is under the control of a high selective pressure, traits associated with reproduction appear to evolve faster than other features. The study of mechanisms involved in the differentiation of sexual behaviors thus constitutes an invaluable tool for understanding of sex differences in general [2], [3].

In many vertebrates, the neural conversion of testosterone into estradiol by the enzyme aromatase plays a key role in the sexual differentiation of brain and behavior [4], [5], [6], [7] and in the activation of masculine sexual behaviors in adulthood [8], [9], [10]. Thus, aromatase has emerged as a potential factor contributing to brain sex differences underlying the activation of male-typical sexual behavior. It was hypothesized that the sexually differentiated expression of this behavior (expressed only by males) results from a differential metabolism of testosterone in specific brain regions. The comparison of aromatase activity (AA) in microdissected brain regions of various species of tetrapods revealed that aromatase is more active in males than in females throughout the hypothalamus and especially in the preoptic area (POA) [11], [12], [13], [14]. In both sexes, AA is reduced to basal levels after gonadectomy, while chronic treatment with exogenous testosterone restores high enzymatic activity [11], [12], [13], [15], [16]. Yet, equating circulating testosterone concentration does not always eliminate the sex difference in enzymatic activity [11], [13], [15], [17], [18]. The relative inability of females to display the male-typical copulatory sequence even when treated with testosterone might thus result from their inability to aromatize testosterone as efficiently as males do. However, other studies, sometimes in the same species, found that exposing both sexes to similar hormonal conditions abolished the sex difference in AA thus casting doubt on the causal link between low AA and absence of male-typical behavior [14], [16], [18], [19].

Also, it was suggested that perinatal steroid exposure of rat pups could reverse the sex specific pattern of AA in parallel with the reversal of sexual behavior phenotype [20]. In one study on quail, prenatal exposure of male embryos to estrogens decreased adult preoptic AA while demasculinizing in parallel copulatory behavior but the same treatment unexpectedly increased AA in females thus raising questions regarding the physiological interpretation of this effect [19].

Since these earlier studies, numerous discoveries have fundamentally affected our understanding of brain aromatase in quail and other vertebrates. The development of aromatase specific antibodies [21], [22] and of *in situ* hybridization procedures [23], [24] allowed an accurate delineation of the populations of aromatase-containing cells and revealed that sex differences in AA are not necessarily correlated with sex differences in the numbers of aromatase-immunoreactive (ARO-ir) cells or the density of the corresponding mRNA (e.g., [13], [14], [15], [24], [25], [26], [27]). Brain aromatase was demonstrated to be present and active both in the soma and in presynaptic boutons [28], [29], [30], [31] raising the possibility that estrogens may be produced and act differently in different subcellular compartments. Finally it was discovered that AA can be modulated by changes in enzyme concentration (control by steroids of the transcription of the corresponding gene) but also, more rapidly, by calcium-dependent post-translational changes (phosphorylations) of pre-existing enzyme molecules [32], [33]. Interestingly, these rapid modulations of AA by calcium-dependent phosphorylations seem to be sexually differentiated [34].

With this new knowledge in mind, we analyzed here in micropunched nuclei the distribution and control by embryonic and adult sex steroids of AA in the quail brain based, for the first time, on the precise anatomical localization of aromatase-expressing cells. These studies establish the high degree of anatomical specificity in the control of AA and reveal the complex causal links between embryonic and adult endocrine environment, local brain AA and sex differences in appetitive and consummatory sexual behavior.

## Methods

### Subjects

A total of 80 Japanese quail (Coturnix japonica) served as subjects (Experiment 1, n = 20; Experiment 2, n = 60). Animals were obtained from a local breeder at the age of 6 weeks (Experiment 1) or as fertilized eggs (Experiment 2). Throughout their life at the breeding colony and in the laboratory, birds were exposed to a photoperiod simulating long days (16 hours light and 8 hours dark per day) and had food and water available *ad libitum*. All experimental procedures were approved by the Ethics Committee for the Use of Animals at the University of Liège (Laboratory authorization number LA1610002; Ethic protocol numbers 683 and 859).

### In vivo treatments

Subjects used in Experiment 1 were sexually mature and gonadally intact. At the age of 8 weeks, males (n = 10) were given 2 copulatory pretests to insure that they were displaying male sexual behavior. All females (n = 10) were laying eggs. The day following the second copulatory test, all subjects were killed by rapid decapitation. Their brain was immediately frozen on dry ice and kept at −80°C until further use.

For Experiment 2, fertilized eggs were set in an incubator at 38°C and 50–60% of relative humidity and injected on day 7 of incubation with either 25 µg of estradiol benzoate (EB, Sigma; 500 µg/ml in sesame oil), or 10 µg of the non-steroidal aromatase inhibitor, Vorozole (VOR; R83842, generously provided by Dr. R DeCoster, Janssen Research Foundation, Beerse, Belgium; 200 µg/ml in 20% propylene glycol/saline solution) or their respective vehicle (number of animals in each group: see figures). Briefly, injections of sterile solutions were performed with a 25-gauge needle targeting in the albumen through the shell at the small end of the egg. The holes were then sealed with melted paraffin (see [4], [35] for detail).

Birds were gonadectomized at 4 weeks post hatch as previously described [4]. At the age of 8 weeks, males and females were subcutaneously implanted with a 20 mm-long Silastic™ capsule (Degania Silicone Ltd., Israel; 1.57 mm i.d.; 2.41 mm o.d.) filled with crystalline testosterone (Fluka). They were then transferred to individual cages. The androgen-dependent cloacal gland area [36] of each subject was regularly measured with calipers (greatest length multiplied by the greatest width in mm^2^) to monitor the androgenic stimulation resulting from these testosterone implants. A month later, all birds were submitted to standardized behavioral tests to assess their appetitive and consummatory sexual behavior (1 or 3 tests respectively). Two days after the last test, all birds were killed by rapid decapitation. Their brain was immediately frozen on dry ice and kept at −80°C until further use. The autopsy determined that all birds were completely castrated and still possessed their testosterone implants.

### Behavioral tests

Appetitive sexual behavior was assessed by the measure of the rhythmic cloacal sphincter movements (RCSM) produced in response to the visual presentation of a female in an aquarium divided into two chambers [37], [38] (see [39] for detail).

To assess consummatory sexual behavior, the experimental subject was introduced into a test arena (60×40×50 cm) that already contained a sexually mature hen. Both birds could then freely interact for 5 min during which the frequency and latency of first occurrence of male sexual behaviors were recorded. The following behavior patterns were systematically noted: neck-grabs (NG), mount attempts (MA), mounts (M) and cloacal contact movements (CCM) (see [40], [41] for description). As NG and M frequencies closely parallel those of MA and CCM, only data relative to MA and CCM will be presented here to avoid redundancy. The behavior frequencies recorded during the three tests were summed for statistical analysis. Only behavioral latencies from the last test were considered.

### Brain dissections

Brain nuclei containing dense populations of ARO-ir cells [22] were dissected by the Palkovits punch technique [42] as previously adapted for the quail brain [43]. Briefly, the brain was sectioned in 200 µm-thick cryostat coronal sections with the plane of section adjusted to the stereotaxic atlas of the quail brain [44]. Sections were mounted on frozen microscope slides and individual regions were then immediately collected by punching them out of the sections with cannulae made of 21G or 18G stainless steel needles (o.d. 0.819 and 1.270, respectively; i.d. 0.514 and 0.838 mm, respectively).

**Figure 1:**
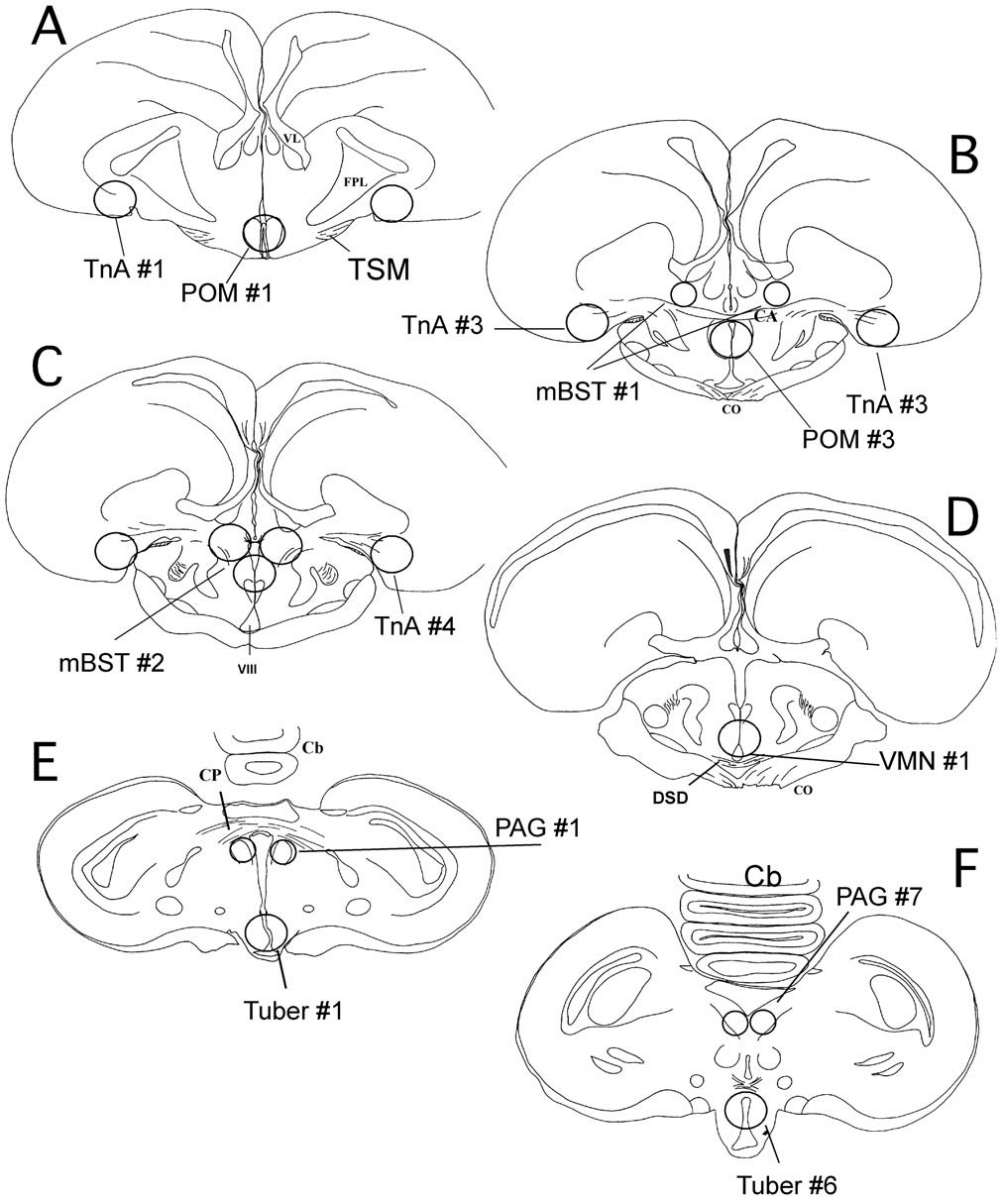
Schematic drawings of coronal sections through the quail brain from anterior to posterior illustrating the areas that were punched to quantify aromatase activity. The circle diameter reflects the diameter of the micropunch needle (o.d. 0.8 or 1.2 mm) used to collect the tissue. Abbreviations: mBST, medial portion of the bed nucleus of the stria terminalis; CA, commissura anterior; Cb, cerebellum; CO, optic chiasma; CP, commissura posterior; DSD, decussatio supraoptica dorsalis; FPL, Fasciculus prosencephalii lateralis (lateral forebrain bundle); Tub, Tuber; PAG, periacqueductal gray; POM, medial preoptic nucleus; TSM, tractus septopallio-mesencephalicus; VIII, third ventricle; VL, ventriculus lateralis; VMN, ventromedial nucleus of the hypothalamus; TnA, nucleus taeniae of the amygdala.

A total of six brain areas (see Fig. 1) were collected: the medial preoptic nucleus (POM), the medial portion of the bed nucleus of the stria terminalis (mBST), the nucleus Taeniae of the Amygdala (TnA), the dorsal edge of the ventromedial nucleus of the hypothalamus (VMN), the tuberal region (Tuber) and the periacqueductal gray (PAG; see Table 1 for detail). Punches were blown out of the needles in a refrigerated 1.5 ml Eppendorf™ tube, immediately frozen on dry ice and kept at −80°C until further use. Slices were then thaw mounted and stained for Nissl bodies with Toluidine blue to confirm the location of the micropunches. This anatomical analysis was performed by the experimenter who was blind to the sex and experimental group of samples and used to confirm that samples had been collected at the appropriate location.

**Table 1:** Diameter of stainless steel needles used to collected tissue and protein content of regions in 200 µm frozen brain sections.

Region	Punch Size^a^(i.d.: µm)	Section	Theoretical protein content (µg)^b^	Averageprotein content(µg)^c^
POM	1×838	1–3	16.5	15.9±0.8
mBST	2×5143×838	34	20.7	22.2±1.2
TnA	2×838	1–4	44.1	45.1±2.0
VMN	1×838	7–12	33.1	32.7±1.1
Tuber	1×838	13–18	33.1	31.9±0.9
PAG	2×514	13–19	29.0	38.7±1.5

Consecutive coronal sections (200 µm) of frozen quail brain were cut beginning at the level of the end of the tractus septopallio-mesencephalicus tractus (section 1) to approximately the level of the third nerve. These sections were numbered following their order of collection.

a All tissues were taken as single midline punches aimed at the collapsed third ventricle, except for the tissue from the TnA and the PAG that consisted of individual left and right punches. For mBST, 2 punches collected the left and right anterior portions (section 3), while 3 punches collected the lateral and medial parts of the posterior portion (section 4; see fig. 1 for details).

b Theoretical protein contents were estimated as follows. The volume of the tissue micropunched was obtained by multiplying the square of the radius of the circle determined by the needle by Pi and by the thickness of the brain slice (For the 514 µm i.d. needle: 3.14×0.26^2^×200 = 0.04 µl; for the 838 µm i.d. needle: 3.14×0.42^2^×200 = 0.110 µl). The theoretical fresh weight of the tissue was calculated assuming that brain tissue has a density close to this of water (0.100 µl = 100 µg). The theoretical protein content was then obtained assuming a protein content of about 5% (2.1 and 5.5 µg/punch for the 514 and 838 µm i.d. needle respectively).

c These data represent the mean ± S.E.M. of micropunches collected in both experiments across treatment groups (n = 75–80).

Tissue punches were homogenized in 120 µl of ice-cold TEK buffer (10 mM Tris-HCl, 1 mM Na-EDTA, 150 mM KCl, pH 7.2) with a glass pestle fitting Eppendorf™ tubes and stored at −80°C until assayed.

### Aromatase assays

AA was quantified by measuring the tritiated water production from [1β-^3^H]-androstenedione (Perkin-Elmer, Specific activity 23.5 Ci/mmol) as described previously and validated for the quail brain [45]. Briefly, equal volumes (50 µl) of tissue homogenates, [1β-^3^H]-androstenedione (100 nM), ice-cold TEK buffer and NADPH (4.8 mM) were incubated for 15 min at 37°C. The reaction was stopped by cooling the samples in an ice bath and adding 0.4 ml ice-cold 10% trichloroacetic acid containing 2% activated charcoal. After centrifugation at 1200 *g* for 15 min supernatants were applied to a Dowex cation exchange resin AG 50W-X4, 100–200 mesh (Biorad, Richmond, CA). Columns were then eluted with distilled water. Optiphase “Highsafe” 3 (Perkin Elmer) was added to the effluent that was then counted for 3 min on a Wallac Winspectral 1414 Liquid Scintillation Counter.

Male or female preoptic-hypothalamic (HPOA) homogenates (n = 3) were assayed as triplicates to serve as internal controls (see below). In addition, an extra tube was assayed for each HPOA homogenate in the presence of an excess (40 µM final concentration) of the potent and specific aromatase inhibitor, Vorozole (see above). These values were pooled and subtracted as blanks to determine the final specific activities. Blank values never exceeded 100 dpm while control samples had radioactivities ranging between 1000 and 4000 dpm. Enzyme activity was expressed in nmol h^−1^ mg protein^−1^ after correction of the counts for quenching, recovery, blank values and percentage of tritium in β-position in the substrate.

All experimental samples were assayed for AA as duplicates run in several assays for each experiment (4 for Experiment 1; 6 for Experiment 2) with the HPOA homogenates used as internal standards. Intra-assay coefficients of variation were always inferior to 7% and inter-assay coefficients of variation were inferior to 5%.

### Protein assay

The protein content of each sample was assayed in 10 µl of homogenate using the commercial Coomassie Plus Protein Assay reagent (Pierce, Rockford, IL). These individual protein values were used to normalize the enzymatic activity of the individual punches. The average protein content per region was almost equal to and correlated with the theoretical protein content (R = 0.93; see Table 1 for estimation details).

### Data analysis

Data of experiment 1 were analyzed by mixed-design two-way ANOVA with sex as the independent factor and brain regions as the repeated measure. All data from experiment 2 were analyzed by 2-way ANOVA with sex and embryonic treatments as independent factors. When appropriate, individual means were compared by post-hoc Fisher protected least significant difference test.

With the exception of a sex difference in favor of females identified in the VMN in the second experiment, no sex, treatment or sex by treatment effect was found to affect protein content in any brain regions studied. In both experiments, enzymatic activities were analyzed before and after correction for protein content (see previous section). Both uncorrected and corrected activities yielded similar statistical results. Because activities expressed as a function of protein content of the sample are corrected for an eventual partial loss of punches during the microdissection and are thus more appropriate, only results corresponding to these corrected activities are presented here.

## Results

### Experiment 1 – Sex differences in AA in the brain of gonadally intact subjects

**Figure 2:**
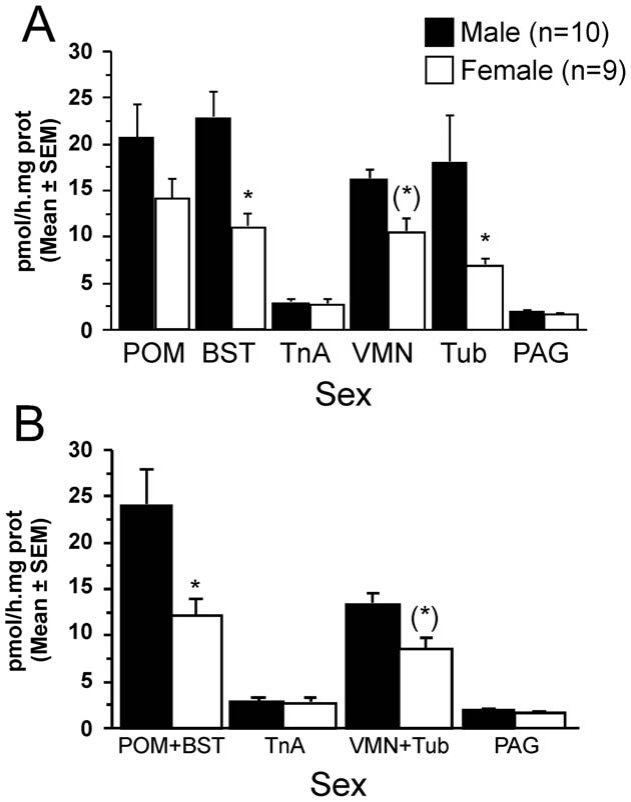
Aromatase activity (mean±SEM) measured in different regions of gonadally intact male and female Japanese quail. **A**. A mixed-design ANOVA with all regions considered individually revealed a significant effect of sex (F_1,17_ = 9.284; p = 0.007) and brain region (repeated factor; F_5,85_ = 20.640; p<0.001) as well as an interaction between these two factors (F_5,85_ = 2.473; p = 0.038). **B**. A mixed-design ANOVA of results after pooling data for POM and mBST as well as for VMN and tuber (MBH) revealed significant effects of the sex (F_1,17_ = 9.407; p = 0.007) and regions (F_3,51_ = 43.392; p<0.001) as well as a significant interaction (F_3,51_ = 4.776; p = 0.005). Individual means were compared by the Fisher Protected Least Significance Difference test. Comparisons between males and females within a given region are reported at the top of the columns as follows: (*) 0.05 <p<0.10; * p<0.05.

The average AA was numerically higher in males than in females in most brain regions (Fig. 2). Accordingly, the analysis of these data revealed a significant effect of both factors and their interaction was also statistically significant (see Fig. 2 legend for detail). Post-hoc analyses focusing on sex differences indicated that AA was significantly higher in males than in females in the mBST and the tuber (Fig. 2A).

Although they encompass different brain structures as defined by Nissl staining and hodological analysis, the ARO-ir cells of the POM and mBST form a continuous cell population. Likewise, the group of ARO-ir cells located at the dorso-lateral edge of the VMN and in the tuber are present without discontinuity throughout the mediobasal hypothalamus (MBH) [22]. A second analysis considering these pooled nuclei as individual entities again identified statistically significant effects of the sex and region as well as a significant interaction. Post-hoc analyses demonstrated that these effects originate from a significant male-biased AA in the pooled tissue from POM+mBST as well as a nearly significant (p = 0.067) male-biased AA in the MBH (Fig. 2B).

### Experiment 2 – Effects of embryonic treatments followed by chronic testosterone treatment on morphology, male sexual behavior and aromatase activity

**Figure 3:**
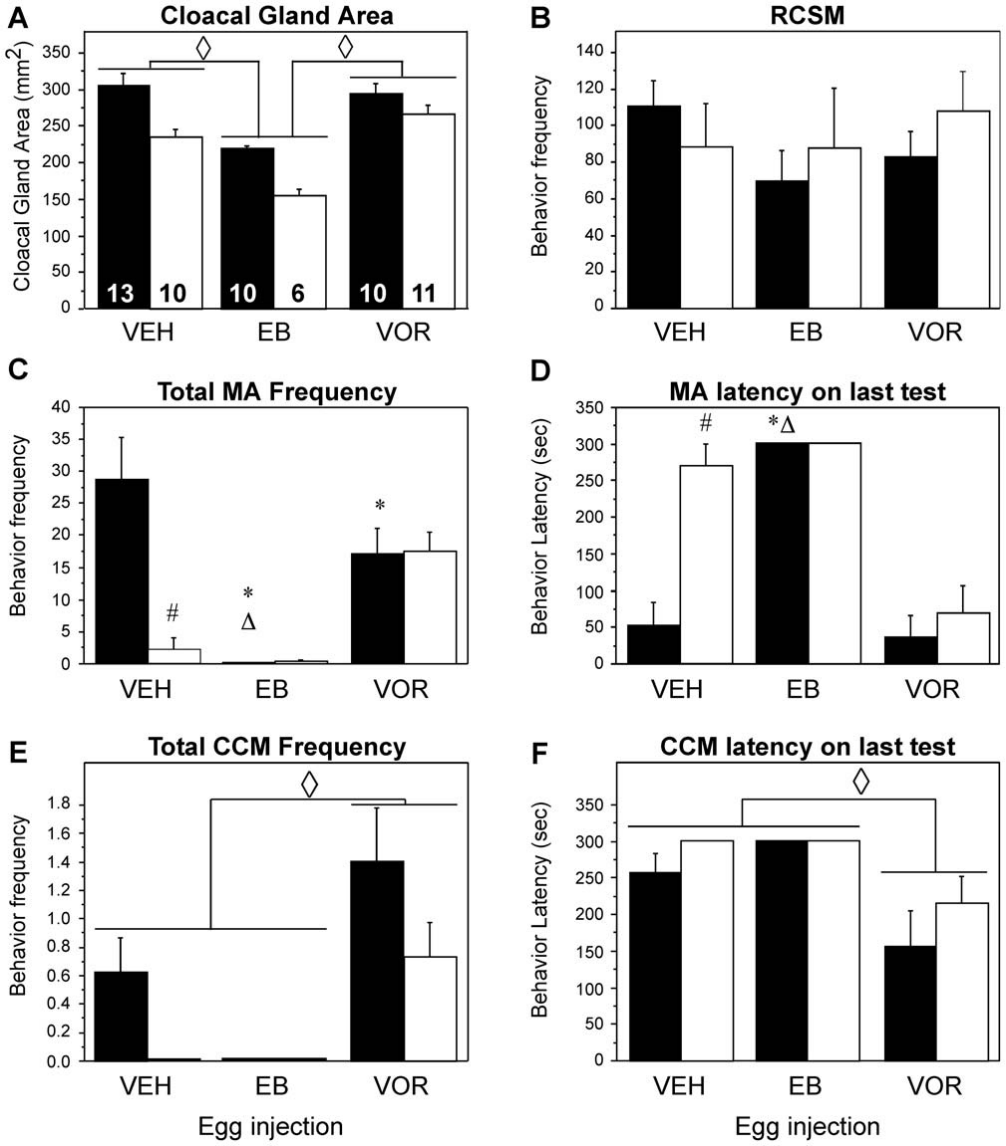
Effect of the embryonic treatments with estradiol benzoate (EB) or Vorozole (VOR) on the cloacal gland area and on different aspects of male sexual behavior of gonadectomized male (black bars) and female (white bars) quail that were treated as adults with exogenous testosterone. All data are means ± SEM. The number of subjects per group is indicated in bars of panel A. **A**. The cloacal gland area was significantly affected by the sex of the birds (F_1,54_ = 26.068; p<0.001) and their treatments (F_2,54_ = 27.798; p<0.001) but there was no interaction between these two factors (F_2,54_ = 1.836; p = 0.169). **B**. The frequency of female-elicited rhythmic contractions of sphincter muscles (RCSM) did not differ between sexes (F_1,53_ = 0.208; p = 0.650) or treatments (F_2,53_ = 0.493; p = 0.613) and was not influenced by the interaction of these two factors (F_2,53_ = 0.817; p = 0.447). **C**. The total frequency of mount attempts (MA) displayed during the 3 copulatory tests significantly differed between sexes (F_1,54_ = 5.879, p = 0.0187) and treatments (F_2,54_ = 7.989, p = 0.009) and an interaction between the 2 factors was detected (F_2,54_ = 6.808, p = 0.002). **D**. The mount attempt (MA) latency on the last copulatory test was also different between sexes (F_1,54_ = 12.104, p = 0.001) and treatments (F_2,54_ = 32.564, p<0.001) and a significant interaction was found (F_2,54_ = 8.464, p<0.001). **E**. The total frequency of cloacal contact movements (CCM) displayed during the 3 copulatory tests significantly differed between sexes (F_1,54_ = 5.106, p = 0.0279) and treatments (F_2,54_ = 10.975, p<0.001) and an interaction between the 2 factors was detected (F_2,54_ = 1.134, p = 0.329). **F**. The cloacal contact movements latency on the last copulatory test was also different between sexes (F_1,54_ = 1.895, p = 0.174) and treatments (F_2,54_ = 8.497, p<0.001) and a significant interaction was found (F_2,54_ = 0.449, p = 0.640). When appropriate, individual means were compared by Fisher Protected Least Significance Difference test. Results are reported at the top individual bars as follows: * p<0.05 compared to vehicle injection (VEH) same sex, Δ p<0.05 compared to Vorozole injection (VOR) same sex, # p<0.05 compared to males same treatment, and ◊ p<0.05 compared to other treatment in both sexes (no interaction in this analysis).

The cloacal gland area was significantly affected by the sex and embryonic treatments of the birds but no interaction between the two factors was found (see details in Fig. 3 legend). Post-hoc analyses indicated that the treatment effect resulted from a reduced area in birds hatched from EB-injected eggs. The gland area was also larger in males than in females regardless of the embryonic treatments.

Sexually mature males (but not females) display rhythmic cloacal sphincter movements (RCSM) when visually exposed to a female [37]. As opposed to gonadally intact females, females chronically treated with testosterone also produce high frequencies of female-elicited RCSM [46]. In this experiment, all subjects were treated with exogenous testosterone and displayed high rates of RCSM with similar frequencies regardless of their sex or embryonic treatment (no effect of the two factors and their interaction, see Fig. 3B).

Male quail readily display copulatory behavior when given the opportunity, while females never exhibit such behavior even when treated with exogenous testosterone in adulthood. In birds, sexual differentiation is driven by the action of ovarian estrogens before incubation day 12: administration of estrogens demasculinizes males while blockade of ovarian aromatization blocks the endogenous demasculinization of females. These effects, however, are only revealed if subjects are treated with testosterone in adulthood to activate the behavior [4], [6], [47]. Accordingly, as illustrated in Fig. 3C–F, male-typical copulatory behavior (illustrated here by MA) was regularly observed in control males but not in control females (2 of these females only displayed 1 or 2 MA that may be interpreted as signs of aggressive behavior). In contrast, VOR-treated birds of both sexes were equally active (9 out of 10 females displayed copulatory behavior as rapidly and frequently as intact males) and sexual behavior was essentially absent in all EB-treated birds (with the exception of one female that showed one NG and MA).

ANOVAs fully confirmed these effects for the frequency and latency of all behavior patterns. They identified significant overall effects of sex (MA frequency: F_1,54_ = 5.878, p = 0.0187; MA latency: F_1,54_ = 12.104, p = 0.001; CCM frequency: F_1,54_ = 5.105, p = 0.028; CCM latency: F_1,53_ = 1.984, p = 0.165) and of treatment (MA frequency: F_2,54_ = 7.989, p = 0.009; MA latency: F_2,54_ = 32.564, p<0.001; CCM frequency: F_2,54_ = 10.975, p<0.001; CCM latency: F_2,53_ = 8.214, p<0.001). A significant interaction between these factors was observed for MA but not for CCM and latency (MA frequency: F_2,54_ = 6.808, p = 0.0023; MA latency: F_2,54_ = 8.464, p<0.001; CCM Frequency or latency: p≥0.32; see Fig. 3 C–F for the detail of post-hoc tests comparing all groups to identify the origins of these interactions). Embryonic treatments followed by testosterone treatment in adulthood thus produced the predicted phenotypes.

**Figure 4:**
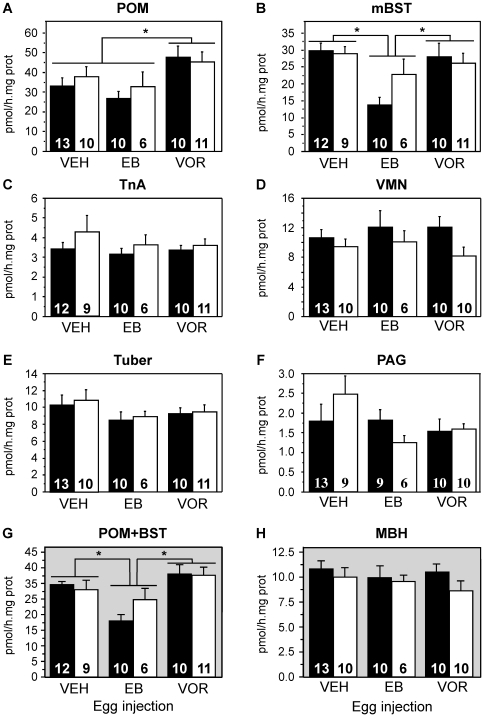
Effect of embryonic treatments with estradiol benzoate (EB) or Vorozole (VOR) on aromatase activity (AA; mean ± SEM) in the brain of adult gonadectomized male (black bars) and female (white bars) quail that were treated as adults with exogenous testosterone. Figures in the bars represent the numbers of final data points available for each group. Results for each nucleus were analyzed by a two-way ANOVA with the sex of the birds (Sex) and their embryonic treatment (TRT) as factors. Interactions (INT) between these factors were also evaluated. Data are illustrated for **A**) the medial preoptic nucleus (POM; Sex: F_1,54_ = 0.382, p = 0.539, TRT: F_2,54_ = 5.093, p = 0.009, Int: F_2,54_ = 0.355, p = 0.703), **B**) the medial bed nucleus of the stria terminalis (mBST; Sex: F_1,52_ = 0.810, p = 0.372, TRT: F_2,52_ = 7.192, p = 0.002, Int: F_2,52_ = 1.870, p = 0.164), **C**) the nucleus taeniae of the amygdala (TnA; Sex: F_1,53_ = 1.408, p = 0.241, TRT: F_2,53_ = 0.791, p = 0.459, Int: F_2,53_ = 0.107, p = 0.899), **D**) the ventromedial nucleus of the hypothalamus (VMN; Sex: F_1,53_ = 4.252, p = 0.044, TRT: F_2,53_ = 0.170, p = 0.844, Int: F_2,53_ = 0.276, p = 0.760); **E**) the tuber (Sex: F_1,54_ = 0.230, p = 0.636, TRT: F_2,54_ = 1.640, p = 0.204, Int: F_2,54_ = 0.030, p = 0.975), **F**) the periacqueductal gray (PAG; Sex: F_1,52_ = 0.039, p = 0.844, TRT: F_2,52_ = 1.758, p = 0.183, Int: F_2,52_ = 1.360, p = 0.266), **G**) the POM and mBST together (Sex: F_1,52_ = 0.475, p = 0.494, TRT: F_2,52_ = 7.901, p = 0.001, Int: F_2,52_ = 1.162, p = 0.321) and **H**) the mediobasal hypothalamus (MBH = VMN+Tuber; Sex: F_1,53_ = 1.428; p = 0.237, TRT: F_2,53_ = 0.419; p = 0.660, Int: F_2,53_ = 0.271; p = 0.764). Origins of the main effects of TRT in the POM, mBST and POM+mBST were analyzed by post-hoc Fisher Protected Least Significance Difference tests. Results are reported at the top of the two columns as follows: * p<0.05 compared to different treatment in both sexes.

Analysis of AA in the POM and mBST revealed significant effects of treatment in both nuclei (POM: p<0.01; mBST: p<0.002) but no effect of sex (p≥0.372) and no interaction between factors (p≥0.164) (see Fig. 4A–B legend for detail of statistical results). Post-hoc analyses identified, however, a different pattern of responses in these two nuclei. In the POM (Fig. 4A), VOR treatment resulted in a higher AA than in control birds and than in EB-treated birds, while in the mBST EB individuals had decreased AA by comparison with both VEH and VOR subjects. (Fig. 4B).

When activities measured in both nuclei were combined, the treatment effect was maintained (F_2,52_ = 7.901, p = 0.001) but there was still no overall effect of sex (F_1,52_ = 0.475, p = 0.494) and no interaction between the two factors (F_2,52_ = 1.162, p = 0.321). Post-hoc tests of the treatment effect revealed that both the control (VEH) and the EB groups were significantly different from the VOR group (Fig. 4G).

In the VMN, AA was numerically lower in females than in males. This effect was close to significance (F_1,53_ = 4.252, p = 0.044). No treatment effect and no interaction of sex with treatment was found.

No effect of sex (all p≥0.16), treatments (all p≥0.18), or interaction (all p≥0.66) was found in the three other brain regions (TnA, Tuber, PAG) or when considering the VMN and tuber as a single population (MBH) (see Fig. 4 for details).

## Discussion

Although sex differences in the brain have been the focus of extensive studies for more than 3 decades, the relationship between sex-related variations in the brain and many behavioral sex differences still remains unexplained. We utilized our detailed knowledge about the distribution of ARO-ir cells [21], [22] and cells expressing the aromatase mRNA [23], [24] to guide our micropunch sampling method. Standard histological procedures such as a Nissl stain are traditionally used to define boundaries of brain nuclei (e.g., [44], [48]). However, decades of chemical neuroanatomical studies demonstrated that specific neurochemical systems do not always map precisely onto these cytoarchitectonic boundaries. This is certainly the case for aromatase [23]. By sampling tissue based on cell populations that express the enzyme, we avoided dilution of active enzyme by a variable amount of tissue that could potentially produce biased results and were thus able to assess enzymatic activity in a more precise manner than previously.

We first confirmed that, in quail, AA is significantly higher in several areas of the male brain as compared to female and determined for the first time the exact localization of these sex differences. We also demonstrated that embryonic manipulations of estrogens affect adult brain AA in parallel with copulatory behavior but do not determine the sex difference in AA that is essentially the result of activational effects of hormones in adulthood. These findings have significant implications for the understanding of behavioral sex differences.

### Distribution of aromatase activity in gonadally intact birds

The present study confirmed and extended the previously described distribution of AA in the quail brain. AA is very high in the POM but much lower in the TnA [14], [43]. The presence of aromatase in the mBST was unknown when previous enzymatic studies [14], [43] were performed (no immunohistochemical or *in situ* hybridization procedure was available at that time to localize the enzyme or its messenger) and AA had not been quantified in this nucleus. Consistent with the fact that ARO-ir cells in POM and mBST form a compact group without discontinuity, AA in mBST was found to be as high as in POM.

Enzyme activity was much higher in VMN and Tuber than anticipated. In previous experiments [14], [43], AA in VMN or Tuber was below 25% of values observed in POM whereas here this ratio was around 90% in sexually mature gonadally intact birds (Experiment 1). This discrepancy could be due to the use of a different assay, associated to a regional difference in estrogen catabolism. The product formation assay (measure of estradiol produced) used previously is indeed sensitive to estrogen metabolism (the product disappears from the reaction tube leading to an underestimation of AA), whereas the tritiated water assay is not. However, it is more likely that the discrepancy results from a difference in the location of micropunches. The population of aromatase cells in the VMN and tuber does not coincide exactly with the boundaries of these nuclei as defined by Nissl staining [22], [24] that guided previous assay studies, while we could now dissect nuclei based on the specific location of ARO-ir cells and of the corresponding mRNA [21], [22], [24].

We also quantified here for the first time AA in the PAG because previous work identified in this nucleus a dense axonal projection originating from preoptic ARO-ir cells while very few immunoreactive perikaya were present at this level [49], [50], [51]. Although aromatase is present and active in the synaptosomal fraction of various brain regions [29], [31], [52], a relatively low enzymatic activity was detected in this nucleus. Additional studies are needed to determine whether this low activity results from a lower concentration of the enzyme in the presynaptic boutons as compared to perikarya or from a lower activity of the enzyme present at this level due to post-translational regulation (see also below).

### Sex difference in AA in gonadally intact subjects

The most robust sex differences affecting AA in gonadally intact birds were found in the mBST and the tuber, while smaller (non significant) differences were identified in the POM and VMN. The sex difference observed in whole HPOA blocks during previous studies [13], [14], [19], [43] thus appears to be mainly explained by the difference existing in the mBST (included in the dissections) and tuber, with a lower contribution of the POM and VMN. Interestingly, all differences identified here are in favor of males. This is in clear contrast with the recent *in situ* hybridization data showing either no sex difference (in the POM and MBH) or a sex difference favoring females (in the mBST) in the expression of the aromatase mRNA [24], [27]. This discrepancy is most likely explained by the existence of a differential regulation of mRNA translation or of enzymatic activity in males and females (see below).

Although tract-tracing combined with aromatase immunocytochemistry indicated that the projection from the ARO-ir cells to the PAG is significantly denser in males than in females [51], no sex difference in AA was detected here in the PAG. This negative result might be due to a sensitivity issue preventing our assay to detect a significant difference at a low average enzyme activity. Alternatively, the enzymatic activity could be differentially regulated in the perikarya and in presynaptic boutons [30].

### Activational and organizational effects of steroids on morphology and behavior

The second experiment confirmed the previously established organizational effects of embryonic estrogens on cloacal gland size and male sexual behavior [4], [6], [47]. Administration of exogenous EB to male embryos resulted in males that were, like females, unable to express male copulatory behavior in response to testosterone. All birds injected with estrogens in the egg also developed a smaller cloacal gland in response to testosterone than control subjects. In contrast, embryonic aromatase inhibition in females conferred them the ability to exhibit, like males, the male-typical copulatory sequence when treated in adulthood with testosterone.

In contrast, these embryonic treatments did not affect one appetitive component of sexual behavior, the frequency of RCSM. Adkins-Regan and Leung recently demonstrated that testosterone treated females respond like males to the visual presentation of another female by frequent contractions of the sphincter muscles [46]. These data suggested that the sex difference in RCSM frequency observed in adult gonadally intact birds (males produce RCSM, females do not) only results from a differential activation by testosterone in adulthood and is not affected by organizational effects of estrogens during embryonic life. This logical deduction, that had never been tested, is formally demonstrated here. Increases (treatment with EB) or decreases (treatment with the aromatase inhibitor vorozole) in estrogens availability that completely sex-reversed the copulatory phenotype had no effect on the frequency of RCSM observed after testosterone treatment in adult birds of both sexes. This aspect of appetitive sexual behavior is therefore absent in females only because they do not have sufficient concentrations of circulating testosterone in their blood. There is no organization by steroids of this behavior during ontogeny.

### Sexual differentiation of aromatase activity and copulatory behavior

The present results also indicate that early estrogen action has long-lasting (organizing?) effects on AA in the POM and mBST. Embryonic treatment with EB decreased (demasculinized) AA in the mBST (a decrease was also observed by comparison with VOR-treated birds in POM), while embryonic aromatase inhibition increased (hypermasculinized?) AA in the POM. When total activity in POM+mBST was considered (allowing more direct comparison with previous studies in which a larger preoptic block was dissected), the inhibition by estradiol was significant as compared to control subjects.

The presumably organizing effects of manipulations of estrogens' exposure in the embryo on AA in POM and mBST are anatomically specific since they are not observed in the hypothalamus itself (VMN and Tuber) nor in other brain regions expressing lower levels of AA. They are paralleled by permanent behavioral effects: a treatment with EB that reduces AA also permanently blocks male copulatory behavior in males, while a treatment with VOR which produces females showing male-typical copulatory behavior in adulthood (VOR blocks their demasculinization by endogenous estrogens) enhances AA in the POM. These correlations suggest the existence of a causal relationship between the developmental effects of estrogens on AA and on male sexual behavior.

However, this organizational action of estrogens on AA does not seem sufficient to explain alone the sex difference in AA seen in gonadally intact birds nor the behavioral sex difference in response to testosterone (males copulate, females do not). Indeed, AA is similar in testosterone-treated males and females that were not exposed to any embryonic treatment but had of course a differential exposure to endogenous estrogens (high in females, low in males [53]). Furthermore, testosterone-treated males mount and copulate in a male-like manner while females do not. It is also established that females treated in adulthood with high doses of estradiol (and thus bypassing the potential bottleneck due to a lower rate of testosterone aromatization) still do not express male-typical copulatory behavior [54]. Together, these data indicate that the sex difference in AA observed in gonadally intact adult quail might potentially contribute to but is not the only cause of the behavioral sex difference.

Alternative mechanisms have to be invoked to explain this behavioral sex difference. It is now established that male and female brains are differently wired [55], [56], [57]. Additionally, sex differences have been observed in the regional brain responses to various social stimuli [58], [59]. In quail specifically, the projection from the POM (a key center in the control of copulation) to the PAG (a mesencephalic pre-motor center) is significantly denser in males than in females [51]. These hodological differences might play a critical role in the control of behavioral sex differences but the present study clearly demonstrates that brain AA cannot be an important factor in the control of these differences.

### Genomic vs. post-translational controls

The preoptic AA in gonadally intact adult quail is invariably higher in males than in females (present study; [13], [14], [15]). In contrast, when subjects are gonadectomized and treated with a same amount of testosterone, this sex difference becomes much less reliable. It is either still present but with a lower amplitude [13], [15] or it disappears completely (present study; [14], [19]). The same results have been observed in other species [11], [16], [17], [18].

A large part of the sex difference in AA observed in gonadally intact birds thus results from a differential induction by testosterone. The residual difference observed in gonadectomized testosterone-treated birds could then reflect organizational effects of estrogens identified in the present study. Why these effects would be apparent in some studies is unclear but might reflect post-translational controls of enzymatic activity as suggested by the comparison with densities of aromatase mRNA and numbers of ARO-ir cells. Indeed, the number of aromatase-positive cells is significantly larger in parts of the POM of intact male Japanese quail than in females but gonadectomy and treatment with testosterone eliminates this sex difference [25], [26]. At the mRNA level, sex differences are even more limited: aromatase mRNA density is similar in the POM of gonadally intact males and females [24] as well as in gonadectomized birds exposed to sex steroids [27]. A reverse sex difference (females>males) was even detected in the mBST in both endocrine conditions [24], [27]. These discrepancies between sex differences in aromatase mRNA, aromatase protein (indirectly assessed by the numbers of ARO-ir cells) and AA suggest that the enzymatic activity can also be regulated by mechanisms that are not related to changes in gene transcription and in enzyme concentration.

Recent experiments have demonstrated that brain AA can be rapidly (within min) regulated [60], [61], [62], [63], [64]. *In vitro* studies indicate that these fast changes in AA result at least in part from calcium-dependent phosphorylations themselves depending on the activity of neurotransmitters such as glutamate [45], [65], [66]. A rapid effect of glutamate on estrogen concentration has also been confirmed in the telencephalon of awake songbirds [63]. Brain AA is rapidly and reversibly affected *in vivo* by a variety of factors such as the sexual interaction with a conspecific or a mild stress and these enzymatic responses are modulated by previous experience [62], [63], [64], [67], [68], [69], [70]. AA measured in all experiments assessing sex differences could thus have been affected in a manner independent of the experimenter by these factors (view of a female, stress and previous history) thus creating the discrepancies that have been reported. In particular, a differential reaction of males and females to these modulatory factors would explain the variability of the sex differences that have been detected. This possibility is reinforced by the recent finding that HPOA homogenates of male and female differentially react to the exposure to phosphorylating conditions (presence of ATP, calcium and magnesium, with or without the calcium chelating agent EGTA) or to kinase inhibitors [34]. The contribution of post-translational controls of AA to the magnitude of sex differences should clearly be investigated.

### Conclusions

The present study provides for the first time an analysis in both sexes of the distribution and regulation by steroids of aromatase activity guided by the anatomical localization of the protein. Manipulations of embryonic and adult concentrations of sex steroid hormones revealed a regional specialization of the genomic mechanisms of regulation of AA. Preoptic AA (POM and mBST) is regulated by a combination of the organizational effect of estrogens with the activational action of testosterone. Importantly, the present data also formally demonstrate that the lower AA in females as compared to males is not the reason why they do not copulate in a male-like fashion as reinstating similar AA in both sexes does not restore the copulatory capacity in females.

## References

[1] Bao AM, Swaab DF (2010). Sex differences in the brain, behavior, and neuropsychiatric disorders.. Neuroscientist.

[2] Becker JB, Berkley KJ, Geary N, Hoampson E, Herman JP (2008). Sex differences in the brain: from genes to behavior.

[3] Hines M (2004). Brain gender.

[4] Balthazart J, De Clerck A, Foidart A (1992). Behavioral demasculinization of female quail is induced by estrogens: studies with the new aromatase inhibitor, R76713.. Horm Behav.

[5] MacLusky NJ, Naftolin F (1981). Sexual differentiation of the central nervous system.. Science.

[6] Balthazart J, Arnold AP, Adkins-Regan E, Pfaff DW, Arnold AP, Etgen AM, Fahrbach SE, Rubin RT (2009). Sexual differentiation of brain and behavior in birds.. Hormones, Brain and Behavior 2 ed.

[7] Roselli CE, Liu M, Hurn PD (2009). Brain aromatization: classic roles and new perspectives.. Semin Reprod Med.

[8] Hull EM, Rodriguez-Manzo G, Pfaff DW, Arnold AP, Etgen AM, Fahrbach SE, Rubin RT (2009). Male sexual behavior.. Hormones, Brain aand Behavior.

[9] Ball GF, Balthazart J, Pfaff D, Arnold AP, Etgen AM, Fahrbach SE, Rubin RT (2009). Neuroendocrine regulation of reproductive behavior in birds.. Hormones, Brain and Behavior 2 ed.

[10] Meisel RL, Sachs BD, Knobil E, Neill JD (1994). The physiology of male sexual behavior.. The Physiology of reproduction 2 ed. New York, vol 2.

[11] Roselli CE (1991). Sex differences in androgen receptors and aromatase activity in microdissected regions of the rat brain.. Endocrinology.

[12] Roselli CE, Horton LE, Resko JA (1985). Distribution and regulation of aromatase activity in the rat hypothalamus and limbic system.. Endocrinology.

[13] Schumacher M, Balthazart J (1986). Testosterone-induced brain aromatase is sexually dimorphic.. Brain Res.

[14] Balthazart J, Schumacher M, Evrard L (1990). Sex differences and steroid control of testosterone-metabolizing enzyme activity in the quail brain.. J Neuroendocrinol.

[15] Balthazart J, Foidart A, Hendrick C (1990). The induction by testosterone of aromatase activity in the preoptic area and activation of copulatory behavior.. Physiol Behav.

[16] Roselli CE, Resko JA (1984). Androgens regulate brain aromatase activity in adult male rats through a receptor mechanism.. Endocrinology.

[17] Roselli CE, Abdelgadir SE, Resko JA (1997). Regulation of aromatase gene expression in the adult rat brain.. Brain Research Bulletin.

[18] Steimer T, Hutchison JB (1990). Is androgen-dependent aromatase activity sexually differentiated in the rat and dove preoptic area?. J Neurobiol.

[19] Balthazart J (1989). Correlation between the sexually dimorphic aromatase of the preoptic area and sexual behavior in quail: effects of neonatal manipulations of the hormonal milieu.. Archives Internationales de Physiologie et de biochimie.

[20] Roselli CE, Klosterman SA (1998). Sexual differentiation of aromatase activity in the rat brain: effect of perinatal steroid exposure.. Endocrinology.

[21] Balthazart J, Foidart A, Harada N (1990). Immunocytochemical localization of aromatase in the brain.. Brain Res.

[22] Foidart A, Reid J, Absil P, Yoshimura N, Harada N (1995). Critical re-examination of the distribution of aromatase-immunoreactive cells in the quail forebrain using antibodies raised against human placental aromatase and against the recombinant quail, mouse or human enzyme.. J Chem Neuroanat.

[23] Aste N, Panzica GC, Viglietti-Panzica C, Harada N, Balthazart J (1998). Distribution and effects of testosterone on aromatase mRNA in the quail forebrain: A non-radioactive in situ hybridization study.. J Chem Neuroanat.

[24] Voigt C, Ball GF, Balthazart J (2007). Neuroanatomical specificity of sex differences in expression of aromatase mRNA in the quail brain.. J Chem Neuroanat.

[25] Foidart A, de Clerck A, Harada N, Balthazart J (1994). Aromatase-immunoreactive cells in the quail brain: effects of testosterone and sex dimorphism.. Physiol Behav.

[26] Balthazart J, Tlemcani O, Harada N (1996). Localization of testosterone-sensitive and sexually dimorphic aromatase-immunoreactive cells in the quail preoptic area.. J Chem Neuroanat.

[27] Voigt C, Ball GF, Balthazart J (2011). Effects of sex steroids on aromatase mRNA expression in the male and female quail brain.. Gen Comp Endocrinol.

[28] Naftolin F, Horvath TL, Jakab RL, Leranth C, Harada N (1996). Aromatase immunoreactivity in axon terminals of the vertebrate brain.. Neuroendocrinology.

[29] Schlinger BA, Callard GV (1989). Localization of aromatase in synaptosomal and microsomal subfractions of quail (Coturnix coturnix japonica) brain.. Neuroendocrinology.

[30] Peterson RS, Yarram L, Schlinger BA, Saldanha CJ (2005). Aromatase is pre-synaptic and sexually dimorphic in the adult zebra finch brain.. Proc Biol Sci.

[31] Rohmann KN, Schlinger BA, Saldanha CJ (2007). Subcellular compartmentalization of aromatase is sexually dimorphic in the adult zebra finch brain.. Dev Neurobiol.

[32] Balthazart J, Baillien M, Cornil CA, Ball GF (2004). Preoptic aromatase modulates male sexual behavior: slow and fast mechanisms of action.. Physiol Behav.

[33] Balthazart J, Ball GF (2006). Is brain estradiol a hormone or a neurotransmitter?. Trends Neurosci.

[34] Konkle ATM, Balthazart J (2011). Sex differences in the rapid control of aromatase difference in the quail preoptic area.. J Neuroendocrinol.

[35] Schumacher M, Hendrick J-C, Balthazart J (1989). Sexual differentiation in quail: critical period and hormonal specificity.. Horm Behav.

[36] Sachs BD (1967). Photoperiodic control of the cloacal gland of the Japanese quail.. Science.

[37] Seiwert CM, Adkins-Regan E (1998). The foam production system of the male japanese quail: characterization of structure and function.. Brain Behav Evol.

[38] Thompson RR, Goodson JL, Ruscio MG, Adkins-Regan E (1998). Role of the archistriatum nucleus taeniae in the sexual behavior of male japanese quail (Coturnix japonica): a comparison of function with the medial nucleus of the amygdala in mammals.. Brain Behav Evol.

[39] Cornil CA, Taziaux M, Baillien M, Ball GF, Balthazart J (2006). Rapid effects of aromatase inhibition on male reproductive behaviors in Japanese quail.. Horm Behav.

[40] Adkins EK, Adler NT (1972). Hormonal control of behavior in the Japanese quail.. J Comp Physiol Psychol.

[41] Hutchison RE (1978). Hormonal differentiation of sexual behavior in Japanese quail.. Horm Behav.

[42] Palkovits M (1973). Isolated removal of hypothalamic or other brain nuclei of the rat.. Brain Res.

[43] Schumacher M, Balthazart J (1987). Neuroanatomical distribution of testosterone metabolizing enzymes in the Japanese quail.. Brain Res.

[44] Baylé J-D, Ramade F, Oliver J (1974). Stereotaxic topography of the brain of the quail.. J Physiol.

[45] Baillien M, Balthazart J (1997). A direct dopaminergic control of aromatase activity in the quail preoptic area.. J Steroid Biochem Molec Biol.

[46] Adkins-Regan E, Leung CH (2006). Hormonal and social modulation of cloacal muscle activity in female Japanese quail.. Physiol Behav.

[47] Adkins EK (1975). Hormonal basis of sexual differentiation in the Japanese quail.. J Comp Physiol Psychol.

[48] Paxinos G, Watson C (1986). The rat brain in stereotaxic coordinates.

[49] Absil P, Riters L, Balthazart J (2001). Preoptic aromatase cells project to the mesencephalic central gray in the male japanese quail (Coturnix japonica).. Horm Behav.

[50] Evrard HC, Harada N, Balthazart J (2004). Immunocytochemical localization of aromatase in sensory and integrating nuclei of the hindbrain in Japanese quail (Coturnix japonica).. J Comp Neurol.

[51] Carere C, Ball GF, Balthazart J (2007). Sex differences in projections from preoptic area aromatase cells to the periaqueductal gray in Japanese quail.. J Comp Neurol.

[52] Roselli CE (1995). Subcellular localization and kinetic properties of aromatase activity in rat brain.. J Steroid Biochem Mol Biol.

[53] Schumacher M, Sulon J, Balthazart J (1988). Changes in serum concentrations of steroids during embryonic and post-hatching development of male and female Japanese quail (Coturnix coturnix japonica).. J Endocrinol.

[54] Schumacher M, Balthazart J (1983). The effects of testosterone and its metabolites on sexual behavior and morphology in male and female Japanese quail.. Physiol Behav.

[55] Gu G, Cornea A, Simerly RB (2003). Sexual differentiation of projections from the principal nucleus of the bed nuclei of the stria terminalis.. J Comp Neurol.

[56] Simerly RB, Swanson LW, Gorski RA (1984). Demonstration of a sexual dimorphism in the distribution of serotonin-immunoreactive fibers in the medial preoptic nucleus of the rat.. J Comp Neurol.

[57] Loyd DR, Murphy AZ (2006). Sex differences in the anatomical and functional organization of the periaqueductal gray-rostral ventromedial medullary pathway in the rat: a potential circuit mediating the sexually dimorphic actions of morphine.. J Comp Neurol.

[58] Martel KL, Baum MJ (2007). Sexually dimorphic activation of the accessory, but not the main, olfactory bulb in mice by urinary volatiles.. Eur J Neurosci.

[59] Halem HA, Baum MJ, Cherry JA (2001). Sex difference and steroid modulation of pheromone-induced immediate early genes in the two zones of the mouse accessory olfactory system.. J Neurosci.

[60] Balthazart J, Baillien M, Ball GF (2001). Rapid and reversible inhibition of brain aromatase activity.. J Neuroendocrinol.

[61] Hojo Y, Hattori TA, Enami T, Furukawa A, Suzuki K (2004). Adult male rat hippocampus synthesizes estradiol from pregnenolone by cytochromes P45017alpha and P450 aromatase localized in neurons.. Proc Natl Acad Sci U S A.

[62] Cornil CA, Dalla C, Papadopoulou-Daifoti Z, Baillien M, Dejace C (2005). Sexual behavior affects preoptic aromatase activity and brain monoamines' levels.. Endocrinology.

[63] Remage-Healey L, Maidment NT, Schlinger BA (2008). Forebrain steroid levels fluctuate rapidly during social interactions.. Nat Neurosci.

[64] Remage-Healey L, Oyama RK, Schlinger BA (2009). Elevated aromatase activity in forebrain synaptic terminals during song.. J Neuroendocrinol.

[65] Balthazart J, Baillien M, Ball GF (2006). Rapid control of brain aromatase activity by glutamatergic inputs.. Endocrinology.

[66] Balthazart J, Baillien M, Charlier TD, Ball GF (2003). Calcium-dependent phosphorylation processes control brain aromatase in quail.. Eur J Neurosci.

[67] Cornil CA, Ball GF, Balthazart J (2008). Sexual experience and rapid effects of copulatory behavior on preoptic aromatase activity.. Soc Neurosci Abstr.

[68] Balthazart J, Cornil CA, Charlier TD, Taziaux M, Ball GF (2008). Estradiol, a key endocrine signal in the sexual differentiation and activation of reproductive behavior.. J Exp Zool.

[69] Cornil CA, Ball GF, Balthazart J (2009). Copulation rapidly modulates aromatase activity in discrete preoptic and hypothalamic regions..

[70] Dickens MJ, Charlier TD, Cornil CA, Ball GF, Balthazart J (2010). Rapid regulation of aromatase activity and the role of stress.. Soc Neurosci Abstr.

